# Precision agricultural robotic sprayer with real-time Tobacco recognition and spraying system based on deep learning

**DOI:** 10.1371/journal.pone.0283801

**Published:** 2023-03-31

**Authors:** Fazal E. Nasir, Muhammad Tufail, Muhammad Haris, Jamshed Iqbal, Said Khan, Muhammad Tahir Khan

**Affiliations:** 1 Advanced Robotics and Automation Laboratory, National Centre of Robotics and Automation (NCRA), Peshawar, Pakistan; 2 Department of Mechatronics Engineering, University of Engineering and Technology, Peshawar, Pakistan; 3 School of Computer Science, Faculty of Sciences and Engineering, University of Hull, Hull, United Kingdom; 4 Department of Mechanical Engineering, College of Engineering, University of Bahrain, Isa Town, Bahrain; Hebei University of Technology, CHINA

## Abstract

Precision agricultural techniques try to prevent either an excessive or inadequate application of agrochemicals during pesticide application. In recent years, it has become popular to combine traditional agricultural practices with artificial intelligence algorithms. This research presents a case study of variable-rate targeted spraying using deep learning for tobacco plant recognition and identification in a real tobacco field. An extensive comparison of the detection performance of six YOLO-based models for the tobacco crop has been performed based on experimentation in tobacco fields. An *F*_1_-score of 87.2% and a frame per second rate of 67 were achieved using the YOLOv5n model trained on actual field data. Additionally, a novel disturbance-based pressure and flow control method has been introduced to address the issue of unwanted pressure fluctuations that are typically associated with bang-bang control. The quality of spray achieved by attenuation of these disturbances has been evaluated both qualitatively and quantitatively using three different spraying case studies: broadcast, and selective spraying at 20 psi pressure; and variable-rate spraying at pressure varying from 15-120 psi. As compared to the broadcast spraying, the selective and variable rate spray methods have achieved up to 60% reduction of agrochemicals.

## Introduction

Precision agriculture has the ability to increase production per acre while also increasing the automation and environmental friendliness of farming operations. Agricultural robotics and automation have been among the core technologies enabling the fourth industrial revolution. They are often regarded as enabling instruments for achieving the United Nations’ Sustainable Development Goals (SDGs) such as no poverty, no hunger, protecting the planet, and life on land. As underpinned in a report published in 2019 by the United Nation Population Division [1], the world population is expected to reach a mark of 9 billion by the end of 2050. These population projections foreshadow rising food insecurity and scarcity issues, particularly in developing countries including Pakistan.

Moreover, several countries have reported alarming residues of agricultural chemicals in soil beds, agricultural products, and even in human blood and adipose tissue [2, 3]. The ongoing study of the exposure of humans to pesticides on a low level is carried out by the Agricultural Health Study [4]. Also, in a developing country like Pakistan, most farmers use conventional broadcast sprayers that spray the entire field including the non-target species. Despite the fact that crops are planted at set distances in a row, the traditional practice of uniform spraying is used, resulting in pesticide overuse. Similar spraying techniques have been used for weeds that often grow in uneven spots. This increases the price, boosts the possibility of agricultural loss, contaminates food, pollutes the environment, and leads to insect resistance to the chemicals applied. Therefore, it is vital to develop smart solutions to lessen the reliance on traditional spraying techniques and manage the risks involved.

Keeping in view the issues face globally, the agricultural sector has now adopted the artificial intelligence (AI) based solutions and have brought a substantial shift in the conventional agricultural practices in the modern world. In the area of spraying technologies, the AI applications are now emerging at high pace with improving learning and analyzing the different condition of crops in real-time. This area leads to the precision spraying techniques that combines the emerging disciplines of robotics, computer vision and artificial intelligence. The integration of these areas make the spraying methods the ability to identify the and differentiate between the crop and weed and apply the desire amount of chemical on the correct plant location. Therefore, agricultural methods that are proactive and more efficient must be employed to ensure an optimal yield by managing crops input in order to prevent a potential food shortage. These input include materials used or added during agricultural production and include agrochemicals like pesticides and herbicides.

This paper addresses the above mentioned issues and provide the solution for developing a vision-guided mobile robot platform in real tobacco fields. A comparative study is also carried out between the deep learning-based YOLO frameworks in terms of robustness, accuracy, and computational speed. Tobacco is grown in more than 100 countries around the world and in Pakistan, it is considered as an important crop as it generates considerable revenue.

Our robotics platform is one step ahead in providing a low cost precision agricultural-based solution for problems encounter in a traditional spraying approaches. Our research study contributes multifaceted aspects, addressing the major technical innovations that include.

1. Design and development of Differential-Drive Mobile Robot (DDMR) for selective-spraying of the row-crops fields. It uses computer vision techniques to replace the conventional manual/broadcast spraying methods with the selective spraying approach implemented by detecting the tobacco plant as a case study. The robotics platform can apply pesticides on 5 rows at a time.
2. Development of the tobacco plant data-set with 6500 tobacco images and 2000 weeds images capture in manifold ambient conditions from local field and can be accessed at https://github.com/Fazalnasirkhan/ARAL-tobacco.
3. Design and development of novel technique for pressure control system where the qualitative and quantitative study is carried for the robot spraying system.
4. An extensive comparative study is carried out for the robot vision system techniques in terms of its accuracy, robustness, and computational speed. Deep learning-based 5 versions of YOLO models are implemented.

## State of the art

Extensive research work about the autonomous robotic platforms developed for weed control has been reviewed by Slaughter et al., [5] and Meshram et al., [6]. In [7, 8], authors have studied the detection of weeds using various sensors and techniques of machine vision, remote sensing, spectral analysis, and thermal images. Khan et al., [9] has developed a GPS guidance-based autonomous agricultural robot. Haar feature-based cascade classifiers have been used to detect three commonly occurring weed types in a maize field. In [10], with the help of a Support Vector Machine (SVM) classifier and features extracted from image histograms, authors have created a crop perception system that determines the leaf density for the quantity of spray required on the plant. The accuracy score of the leaf density classifier varies from 80% to 85%.

A number of studies exist in literature on vision and AI based site-specific agricultural spraying. The work reported in [11] presents treatment based on the plant density and foliage shape. For autonomous spraying systems in greenhouses, the work published in [12] has employed a 270-degree laser scanning sensor to find targets with complicated shapes. In [13], a human-robot collaborative strategy was developed for target-specific spraying using a robotic platform in a complex environment. The proposed method reduces the amount of sprayed material by 50%. Likewise, in [14] a semi-autonomous agricultural sprayer robot has been developed with particular emphasis on human-robot interaction. The human operator has to manually select targets, e.g., grape clusters, using an input device such as a mouse, Wiimote, or digital pen to be sent to the teleoperated spraying robot. Adamides et.al., 2017 [15] have also developed a teleoperated agricultural robotic system that uses different versions of human-machine-interfaces (HMI). For a set of dedicated inputs (keyboard vs. gamepad), various output devices (screen vs. head-mounted display), and single view feedback vs. multiple views, several methodologies have been used and comparisons have been made. Target (grape clusters) detection and identification under varying lighting circumstances, however, has been left as a future work. Research advancements and innovation in the field of agricultural robots were examined by Bechar and Vigneault in [16]. In order to complete tasks in complicated environment, the work demands the use of intelligent systems. Another thorough review in [17] highlights site-specific weed management strategies used in agriculture. The study identifies knowledge gaps and suggests spraying techniques that can adjust pesticide mixture depending on the weed species present in the field. For weed control, they have also stated a great need for open-access annotated image data.

A detailed discussion of the integration of sensor technologies like 3D camera and multi-spectral imaging with artificial intelligence-based decision algorithms were made in [18]. Technical and economic assessments are presented, and the control level of various spraying techniques is investigated. This analysis also shows the economic aspects of the sprayer robot platform in terms of materials and labor savings. The general categories of the sprayer system include: on-off nozzles sprayers, air-blast sprayers, and canopy optimized distribution sprayers. In [19], an optimized flow rate value of the sprayer was achieved using a machine learning-based vision system. The size of the plant and its matching canopy size are computed once the crops and weeds are classified. The authors experimented using several combinations of image features, including Hu moments (to measure shape), edge-oriented histogram (to detect the edges of the plants), and Haralick features (to quantify texture), and color historgram (to measure the distribution of colors). The Random Forest machine learning algorithm was found to be capable of identifying plants from weeds with 95% accuracy. A frame rate of 17.4 has been achieved which is suitable for spraying applications. Similarly, Cheng et. al., [20] have implemented a feature-based learning method that detects and differentiates weeds from rice plants. The target plant’s leaf tip, or the point of interest, is found using a Harris Corner detection technique. Around 24 features (belonging to the colour and texture categories) of the surrounding area were then extracted and fed to the machine learning algorithm, specifically decision trees, support vector machines, and naive Bayes. An unsupervised method (density-based spatial clustering of applications with noise) was also used on identify clusters in large spatial data and to remove the false positive Harris points.

Moreover, in a site-specific crop management procedures that uses the different sources of information like Near-infrared (NIR) spectroscopy, the residual neural network (Resnet) are used for tobacco classification and the quantitative analysis of the contents of the tobacco leaves are perform using the Long Short-Term Memory (LSTM) network in [21–23]. These analysis provide the bases and provide on time decision making process for the site-specific selective spraying.

Since the breakthrough work in 2012 [24] in which deep learning was demonstrated to outperform the cutting-edge computer vision approaches for object detection, deep learning has fundamentally changed the field of artificial intelligence.

## System description and hardware organization

The five main modules of the work are introduced in this section. The first two sections cover the hardware of the system. This is followed by the visual perception module, which employs a deep learning approach to recognize and classify plants in real time, and also locates them in World coordinates based on the camera model. A vision-based navigation system, as well as a novel pressure and flow control system, are then presented.

### Field robot subsystems overview

The current big trend in precision agriculture is the deployment of robots and electric vehicles that are powered by renewable energy. They provide best tools and practices to address issues faced by the agricultural industry, including population expansion, rising fuel prices and their impact on the environment, labour shortages, and climate change. Additionally, a fully electric and digital system must always be present in the agricultural field in order to put industry 4.0 technologies, such as 4G/5G connectivity, artificial intelligence, blockchain, and the Internet of Things (IoT), into operation. The fundamental prerequisites to progressing towards smart farming and Agriculture 5.0 are provided by robotic platforms and solar-powered electric vehicles.

Advantages offered by electric sprayers as compared to traditional methods have been summarized in [10]. As mentioned, despite their existing limitations such as limited battery power and the challenge of achieving a sufficient level of ingress protection for field conditions, electric vehicles supplemented by solar energy harvesting will revolutionize the field of precision agriculture in the near future.

An autonomous agricultural field robot has been developed at the Advanced Robotics and Automation Laboratory (ARAL) [25], a lab that is a part of the National Center of Robotics and Automation (NCRA) [26], in Pakistan, to test the effectiveness of the developed deep learning based spraying system. The effective features of the robot designed at ARAL are mentioned in Table 1. The robot navigates autonomously between crop rows in the field, classifies crops and weeds in real-time, and sprays pesticides accordingly. The real-field performance of the robotic platform was evaluated by conducting several experiments at Mardan Tobacco Research Station, Khyber-Pakhtunkhwa, Pakistan (Coordinates 34°, 12′, 1.98″, 72°, 0′, 4.633″ East), with special permission granted by the secretary Pakistan Tobacco Board, Hayatabad, Peshawar 25000, Khyber-Pakhtunkhwa, Pakistan.

**Table 1:** Agricultural sprayer robot specifications.

Features	Descriptions
Length, Width, Height	60, 32, adjustable height 26-38 inches
Weight(unloaded)	60 kg
Spraying boom width	Adjustable width 56-130 inches
Chemical storage capacity	90 liters
Numbers of rows coverage	5 rows with adjustable width 12-36 inches
Travel speed	upto 2.2 *ms*^−1^
Drive system	Differential drive 24V DC brush motors
Pressure control system	Closed-loop cascaded control system
Vision system	Deep-learning based YOLOv5
Wheels dimensions	Diameter 14 inches
Power source	12V DC lead-acid battery

To keep the design modules, three subsystems have been developed: (1) the Perception System uses images acquired by a camera to recognize the object of interest using a deep learning algorithm, (2) the Navigation System allows the robot to navigate autonomously or semi-autonomously (using remote controlled) to follow the crop rows, and (3) to spray insecticides in a targeted manner, the Spraying System employs a set of nozzles with adjustable spacing between them.

**Fig 1:**
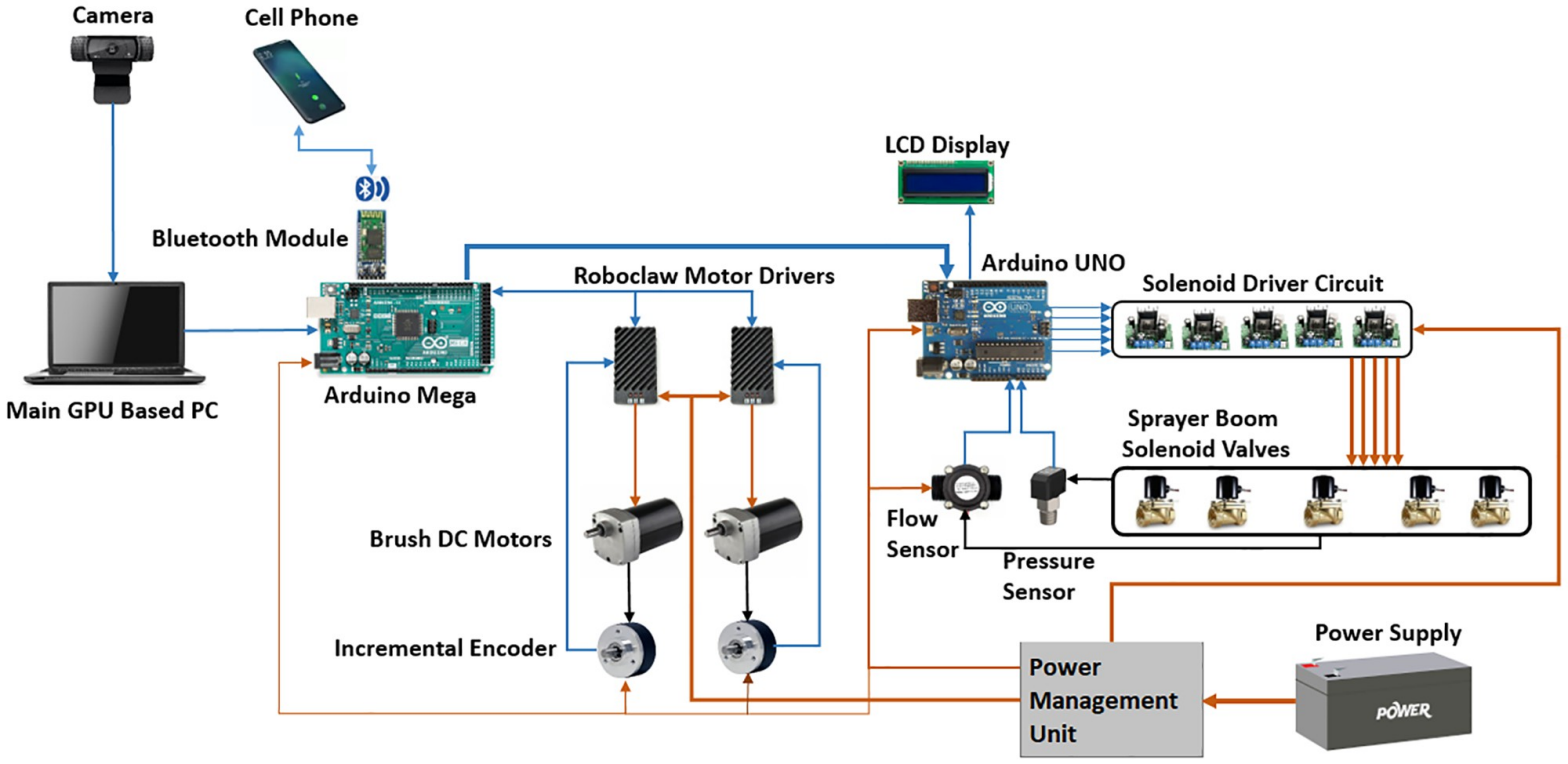
System architecture of precision agricultural spraying robot.

The sprayer robot’s system architecture is shown in Fig 1. An extendable pole (1.5m to 2.10m) from ground level, is fixed on the front of the vehicle that holds cameras. The camera is a Logitech C922 Pro HD camera that can record video up to 1080p at 30fps with a 78-degree field of view. It is used to feed the visual information from field to the AI model running on the onboard PC. The PC used for this experiment has specifications listed in Table 2.

**Table 2:** Computer specifications.

Features	Descriptions
CPU	Intel(R) Core(TM) i7-10750H CPU @ 2.60GHz (64-bit)
RAM	16GB
GPU	NVIDIA GeForce RTX 2070 8GB
Operating System	Microsoft Windows 10 (64-bit)

The spraying system comprises of a telescopic boom with five normally closed solenoid valve nozzles fixed along its length. The distance between the solenoid nozzles valve can be adjusted by sliding the boom links along its lengths (inward or outward). Preemptive spraying (of insecticides/herbicides) is achievable with the help of the developed low-cost smart precision solar-powered robot equipped with embedded AI. Since each solenoid nozzle valve can be independently controlled, the spraying process can be more effectively controlled using the output from the deep learning model that accurately detects and classifies crops and weeds in real-time and subsequently sprays precisely on the desired target plant only.

### Crop perception system

The crop perception subsystem acquires images in real-time and uses a trained deep learning model to detect the crop (tobacco plant). Once the desired plant has been detected, it needs to be localized in the image plane. The corresponding world coordinates of the plant’s centroid are then extracted using the camera model. The output information is then sent to the Spray control subsystem which activates the relevant solenoid.

The most crucial part of any deep learning based visual recognition system is the dataset. It has to be rich enough to be used for training. Different lighting conditions, different growth stages of plants, and different views are all important factors that need to be considered while generating a dataset. A custom dataset of tobacco plants was created for the work discussed here, and it has been made publicly available [27] for the benefit of the research community.

#### Crop detection and classification

Object detection is a widely used computer vision approach for identifying, locating, and labeling individual items in an image or video. The important decision in designing objection recognition system using machine learning techniques is whether to use the traditional machine learning algorithms (e.g., Support Vector Machines, Naive Bayes, k-means clustering, or decision trees) or use modern deep learning algorithms. The former approach involves the feature engineering step which may or may not produce satisfactory results for the object at hand. Previously, the authors have published results where different features and algorithm combinations were tried to reach at the best performing option. Interested readers are invited to see the reference [19].

One of the main problems encountered with traditional machine learning approach has been locating multiple objects in the same image. The popular sliding window approach is generally used to perform classification at large number of locations and scales. However, doing so is computationally expensive, and the cost increases as the resolution and window count fed into the classifier grow. In fact, applications like pest detection and counting fall within this category. As an alternate option, Convolutional Neural Networks (CNN) are good at both localization and detection and have shown promising results for spatially structured data including images. By combining operations such as convolution, pooling and fully connected layers, CNNs can classify local regions in an image at multiple scales and locations.

**Fig 2:**
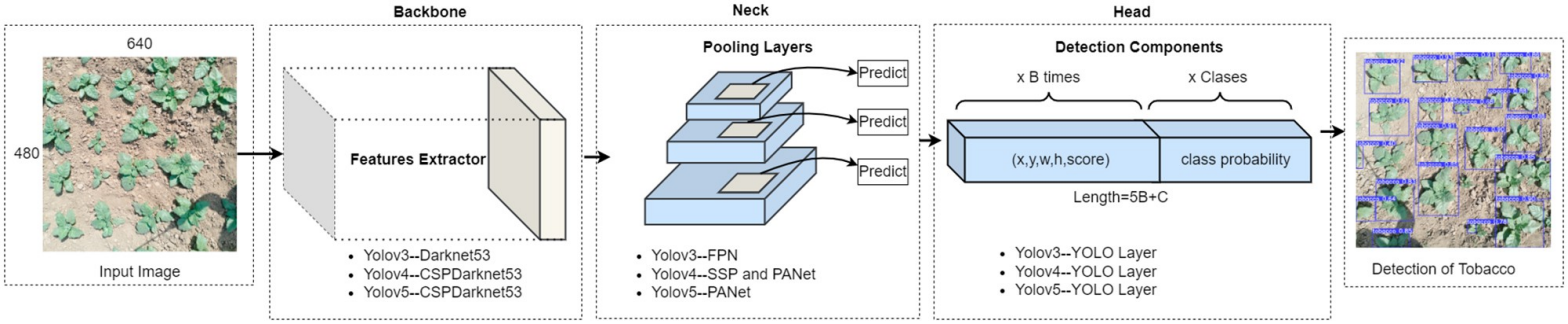
Architecture of YOLO-based object detection model.

One popular CNN is You Only Look Once (YOLO) which has been experimentally found to be robust and well-suited for the problem at hand due to its global approach (as compared to sliding window), high accuracy and better speed. These advantages makes them perfect for real-time deployment as it is a single stage, proposal-free object detector. Overview of the YOLO architecture of version 3,4 and 5 is shown in Fig 2.

YOLO, unlike other previously developed object detection algorithms, addresses object detection as a single regression problem, circumventing the region proposal, classification, and duplicate elimination pipeline [28]. Although YOLO can be deployed in a variety of frameworks, Darknet is the most preferred one for YOLOv3 and YOLOv4 version, while YOLOv5 version is deployed in pytorch framework. Images are downsized to a reduced resolution in YOLO algorithms, and then a single CNN is run on the images, yielding detection results based on the model’s confidence threshold. The sum of square error was reduced in the first version of YOLO (loss function). This optimization improves detection speed but reduces accuracy compared to current object detection models [28]. On-line data augmentation is used in YOLO to improve model robustness in object detection in various contexts by increasing the unpredictability of the input data. YOLO models have been used in a variety of applications where quick detection is required, including pedestrian detection [29], license plate detection [30], and automatic fabric defect detection [30]. Fruit detection [31–34], crop disease diagnosis [35], and weed and pest detection [36] are all examples of YOLO applications in agriculture. Since 2016, multiple versions of YOLO have been released, demonstrating that the algorithm is always improving. Furthermore, each primary version was offered as a full model as well as miniature variants, which had fewer layers and were faster than the full version.

In YOLOv3 Darkent-53 used as feature extractor to extract key features from input image using Convolution layers. It used Featured pyramid netword(FPN) as a neck. The role of neck in YOLO is to give proportionally sized feature maps at multiple levels, in a fully convolutional fashion and the head is composed of Yolo layer which composed of a vector containing bounding box coordinates: width, height, class label, and class probability as shown in Fig 2.

In April 2020, YOLOv4 was launched, with numerous improvements over YOLOv3. CSP Darknet-53 was used to create YOLOv4. The input features are divided into two categories using cross-stage partial connections (CSP): One group is processed by the convolutional layer, while the other bypasses it and is included in the input for the next layer [37]. In the mosaic, an augmented image is generated by combing four input images in a specific ratio. In cutmix, a new image is created using parts of input images. YOLOv4 has more layers compared to the previous versions. The YOLOv4 models use a Complete-IoU loss function to optimise overlap area, centre point distance, and aspect ratio of predicted bounding boxes [38]. The number of layers is reduced in YOLOv4-tiny, and only two YOLO classifiers are employed (both with three anchor boxes).

YOLOv5 was released in May 2020 from Ultralytics LLC (Los Angeles, CA—USA) and and it has been widely embraced by the deep learning community [39]. The key advantage of YOLOv5 is that it is written in Python rather than C language. PyTorch is YOLOv5’s native framework, which enables for quicker training. YOLOv5 offers speedy detection with the same accuracy as YOLOv4 [40] in terms of performance measures. Similar to earlier versions, YOLOv5 was released in various sizes (*s*, *m*, *l*, and *x*) with varying detection accuracy and speed.

YOLO model predictions for each image are encoded as *S* × *S* × (5*B* + *C*) tensor. Here *S* is the number grid cells, *B* is the number of anchor boxes and *C* is the number of classes. For this study, we have used 13 × 13 grid as our target object (tobacco) is a medium-sized object and 13 is an optimal value for the number of grids. Increasing this number further makes the model computationally expensive while a small value reduces the model accuracy. The number of anchor boxes (*B*) is three as it controls the number of objects detected in each grid cells. The model detects a single class (i.e., tobacco), therefore, *C* = 1. In summary, the trained model predictions for tobacco class are encoded as a 13 × 13 × 16 tensor.

### Crop localization and zone formation

After detecting and locating the object in the image frame, its position in image frame is transformed to the global coordinate frame. The central camera is used to observe the imagery information of the crops area covered by boom width. The inverse pinhole model was used to locate the position of the region of interest. The extrinsic and intrinsic parameters of the camera are determined to validate the camera model. These parameters are given in Table 3.

**Table 3:** Intrinsic and extrinsic parameters of camera.

Intrinsic Parameters		Extrinsic Parameters	
Focal Length, *f*	3.3mm	Height, *h*_*c*_	2m
Pixels Size, *ρ*_*x*_,*ρ*_*y*_	*μ*m	Forward Angle, *φ*	18°

**Fig 3:**
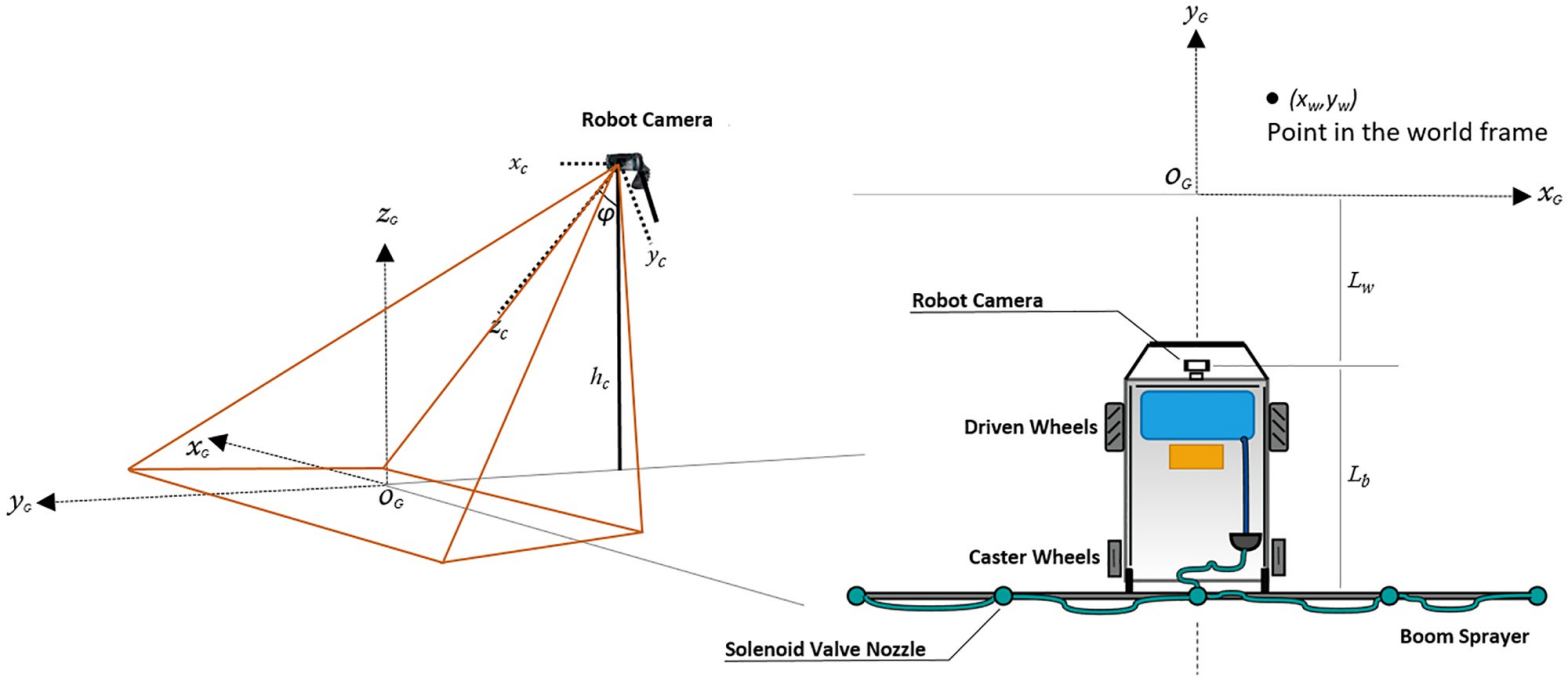
Object coordinates transformation from camera frame to world frame.

The camera is mounted on the front pole at a height, *h*_*c*_ from the ground level and makes an angle *φ* with the vertical axis as shown in Fig 3. Using the camera model [41] the coordinates of the image frame (*x*_*c*_, *y*_*c*_) are translated in global frame, (*x*_*G*_, *y*_*G*_).

Corresponding to a world point, $\tilde{P}={\left[X,Y,Z,1\right]}^{T}$, a point in the image-plane, $\tilde{p}={\left[\tilde{u},\tilde{v},\tilde{w}\right]}^{T}$, can be represented as,

$$\begin{array}{c} \tilde{p}=M\tilde{P} \end{array}$$ (1)

where *M* is the camera matrix, given as

$$\begin{array}{c} M=\underset{\text{intrinsic}}{\underset{︸}{\left[\begin{array}{ccc} f/\rho_{x} & 0 & u_{0}\\ 0 & f/\rho_{y} & v_{0}\\ 0 & 0 & 1 \end{array}][\begin{array}{cccc} 1 & 0 & 0 & 0\\ 0 & 1 & 0 & 0\\ 0 & 0 & 1 & 0 \end{array}\right]}}\underset{\text{extrinsic}}{\underset{︸}{{\left(T_{C}^{G}\right)}^{-1}}} \end{array}$$ (2)

The intrinsic part includes parameters such as focal length *f*, pixel sizes (in micro meters), (*ρ*_*x*_, *ρ*_*y*_), are the horizontal and vertical physical dimensions of the CCD sensor, respectively, and the principal point (*u*_0_, *v*_0_). The extrinsic part comprises of a homogeneous transformation matrix, $T_{C}^{G}$, that relates the camera frame *C* to the world frame *G* and is given as,

$$\begin{array}{c} T_{C}^{G}={\left[\begin{array}{cccc} & R_{C}^{G} & & t_{C}^{G}\\ 0 & 0 & 0 & 1 \end{array}\right]}_{4\times 4} \end{array}$$ (3)

where

$$\begin{array}{c} R_{C}^{G}=[\begin{array}{ccc} 1 & 0 & 0\\ 0 & \text{cos}(\varphi +180) & -\text{sin}(\varphi +180)\\ 0 & \text{sin}(\varphi +180) & \text{cos}(\varphi +180) \end{array}] \end{array}$$ (4)

$$\begin{array}{c} t_{C}^{G}=[\begin{array}{c} 0\\ -h_{C}\text{tan}\varphi \\ h_{C} \end{array}] \end{array}$$ (5)

**Fig 4:**
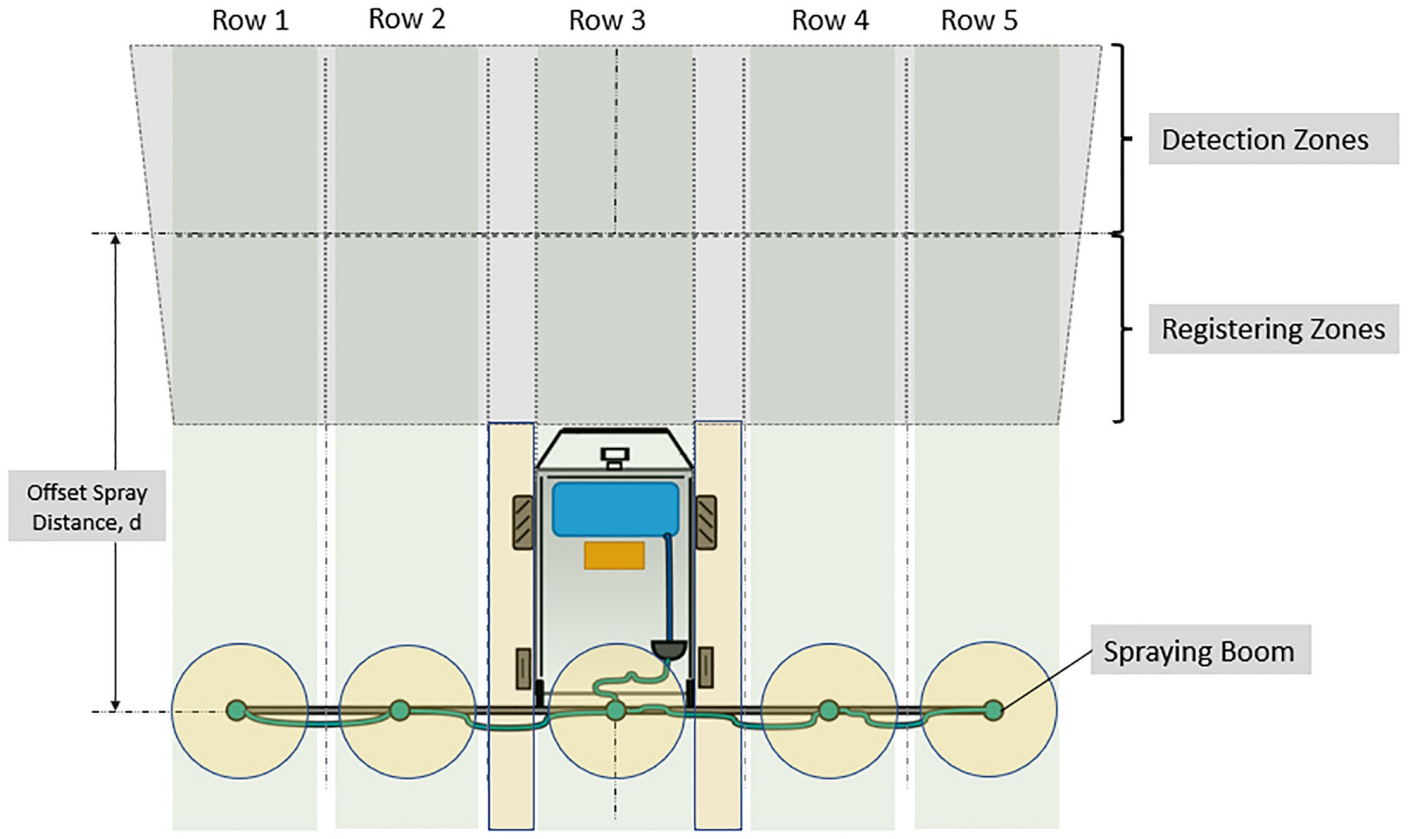
Robot camera field of view with spraying zones formation.

The schematics view of robot and its boom sprayer is shown in Fig 4. The field of view of camera that covers the boom length divides the spraying area into five spraying control zones.

The origin of the world frame is at the middle of the boom length along the x-axis. Based on the position of each plant patch along the x-axis, the nearest zone of the solenoid-valve is assigned for spraying as explained in below section.

#### Distance-based spraying

In this study, a distance-based spraying method is employed to apply pesticides for targeted spraying while synchronizing the image acquisition, processing, and nozzle actuation task. Each identified plant that fills the image frame has its own unique local *x* and *y* coordinates. The global position is determined using the camera model and transformation matrix described in the preceding section.

The camera Field of View (FOV) is divided into 10 zones (2 rows and 5 columns) as shown in Fig 4. The upper row has five detection zones each covers 20 × 42 inches of land area. As the vehicle moves forward, the plant first appears in the detection zones. The plant’s address, *P*_*j*, *n*_ and size in terms of its longitudinal length in forward direction, *L*_*j*, *n*_ are measured. Where, the subscript *n* and *j* represent the *n*th plant in *j*th crop row, respectively. Each crop row is covered by one of five nozzles on the boom (Fig 4). The width of each row (*X*_*i*, *min*_ < *x*_*G*_ < *X*_*i*, *max*_, with *X*_*i*, *min*_, *X*_*i*, *max*_ being the lower and upper limits of the global *x*_*G*_ coordinate) is decided based on the number of nozzles mounted on the sprayer.

At time the plant crosses the detection zone, the crop’s row address, *P*_*j*, *n*_ and a distance from boom, *d*_*j*, *n*_ are registered to the spraying database. This distance is measured from the reference point (*y*_*G*_ = 0) in the global frame to the corresponding nozzle on boom. The distance, *d*_*j*, *n*_ is measured in real-time using the encoders mounted on both wheels. The spray nozzle is triggered by satisfying the condition given in Eq (6).

$$\begin{array}{c} z_{j}=\{\begin{array}{c} ON,\;\text{if}\;\;d_{j,n}<=\frac{L_{j,n}}{2}\\ OFF,\;\text{if}\;\;d_{j,n}>\frac{L_{j,n}}{2} \end{array} \end{array}$$ (6)

Where, *z*_*j*_ = 1, 2, …5 is the nozzle assigned to the *j*_*th*_ crop row for spraying the *P*_*j*, *n*_ plant. This nozzle will remain open for the amount of time given by the Eq (7).

$$\begin{array}{c} \begin{array}{c} T_{j,n}=\frac{L_{j,n}}{v} \end{array} \end{array}$$ (7)

The amount of spray-time, *T*_*j*, *n*_ depends on the longitudinal length, *L*_*j*, *n*_ of the plant and the forward speed, *v* of the robot.

### Spraying control system

Pesticides were applied to crops using hollow cone brass nozzles to perform post-emergence selective spraying. It has a flow rate of 0.3 Gallons Per Minute (GPM) and a cone angle range of 43–120 degrees. To spray the targeted plants, a two-way normally closed type of solenoid valve is used in direct-acting mode. These solenoid valves use a 12V DC power source and can sustain pressures of up to 290 psi. The spraying liquid is transported from a 90-liter chemical tank to the solenoid valves using a 12V, 8A DC diaphragm pump. The pump has a 1.6 GPM flow rate and a 120 psi pressure capacity. The onboard embedded controller adjusts the pump input voltage using a 12V 43A motor driver. The implemented cascaded control system uses the boom pressure and rate of changes in pressure as a feedback to control the PWM signal of the motor driver.

Pressure-based control systems are typically used in agricultural spraying to maintain a steady pressure in the spray boom. The change in pressure during spraying applications is undesirable because it would lead to an unequal distribution of insecticides across the area. Drift occurs during the spraying process as a result of fluctuations in droplet size caused by pressure changes.

#### Pressure and flow control system design

The major purpose of the cascaded control technique is to reject the effect of disturbances on the system’s response. Our solution uses a cascaded Proportional–integral-derivative (PID) controller to regulate the boom sprayer. Although solenoid valves are used to open and close the flow of chemicals, the pressure in the system acts as the control variable.

The main control loop, known as the outer loop, regulates the primary variable, or pressure in the boom system. The system’s pressure is used as feedback and serves as a reference point for the inner loop. The inner loop’s secondary controller regulates how quickly pressure changes occur. In cascaded controllers, the inner loop responds more quickly than the outer loop and is located nearer the disturbance’s source. This enables the inner loop to respond quickly in order to regulate the rate at which pressure fluctuations caused by SV opening or closure are occurring. The Eq (9) gives control law of the outer loop.

$$\begin{array}{c} \begin{array}{c} u_{o}=C_{p}\left(P_{ref}-P\right) \end{array} \end{array}$$ (8)

Where, *P* is the actual boom pressure measured and *P*_*ref*_ is the reference operating pressure set by the user. The *C*_*p*_ and *u*_*o*_ are the gain and control signal (set point for secondary controller) of the main controller respectively. The inner loop uses the rate of change of the pressure as feedback and *u*_*o*_ as a reference point to develop a set point for the secondary proportional controller with a proportional gain *C*_*v*_. The inner loop governing equation is given by Eq (9) and the accumulative control action is calculated as a PWM signal and is given by Eq (10).

$$\begin{array}{c} \begin{array}{c} u_{i}=C_{v}\left(u_{o}-\dot{P}\right) \end{array} \end{array}$$ (9)

$$\begin{array}{c} \begin{array}{c} u=C_{v}\left(C_{p}\left(P_{ref}-P\right)-\dot{P}\right)+C_{i}\int \left(P_{ref}-P\right)dt \end{array} \end{array}$$ (10)

In addition to the cascade controller’s ability to reject disturbances up to a point, the disturbance attenuation function is designed to strengthen the pressure control system’s ability to reject disturbances and more effectively achieve the desired pressure in the system.

**Fig 5:**
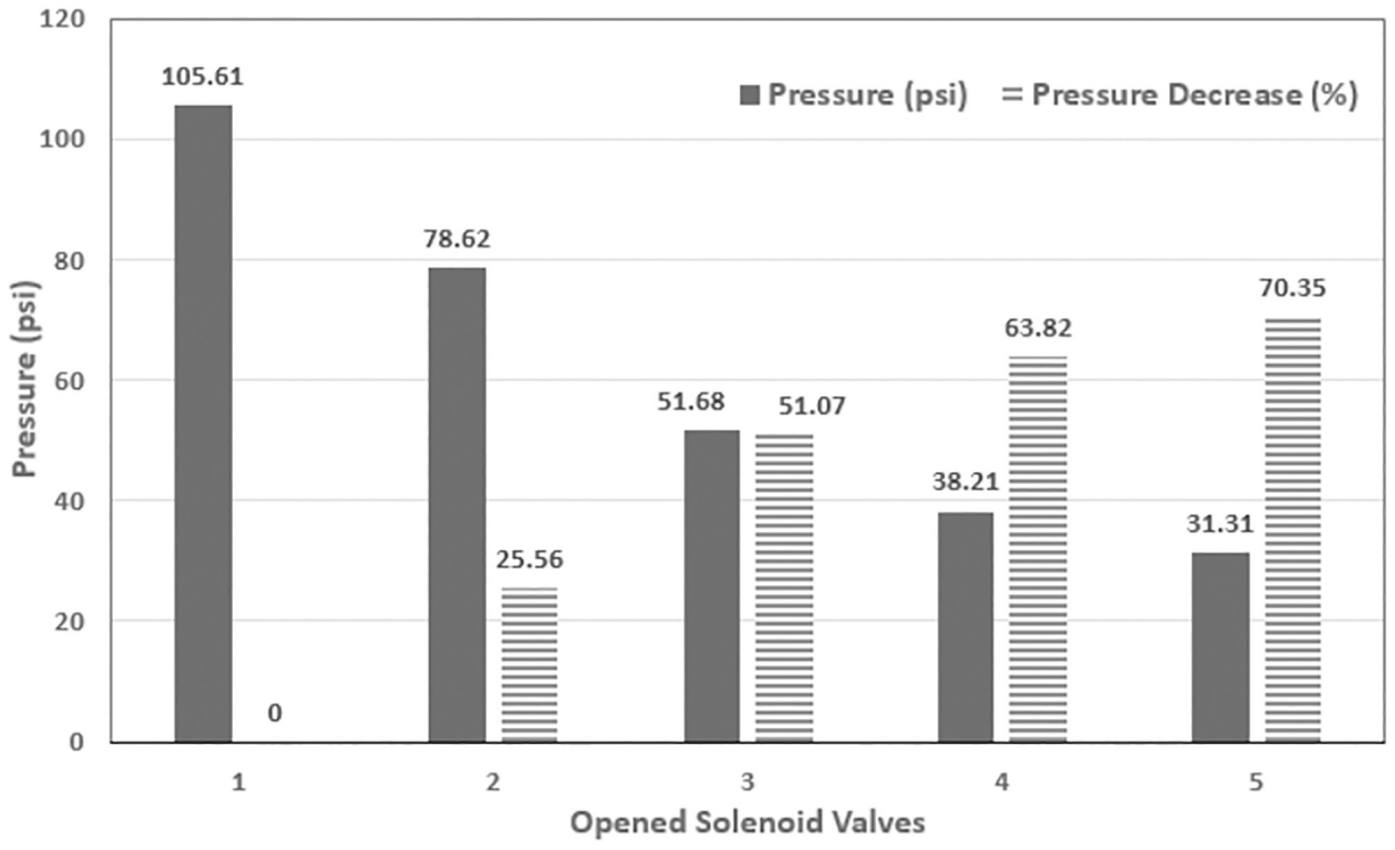
Pressure response with varying numbers of active SVs.

Pressure changes abruptly when the direct-acting type solenoid in the boom sprayer is activated. These disruptions have a significant impact on spray quality and uniformity in pressure-based control systems like agricultural sprayers, where fluid system’s response time is often slower. As a result, they must be avoided. This can be observed in Fig 5 which plots the absolute and percent decrease in pressure values versus the number of open solenoid valves when the pump is running at full duty-cycle.

The input of the disturbance model is the numbers of active solenoid valves, *N_n_* = 1, 2, 3 … 5 while the output is the corresponding signal, *d*_*n*_ fed to the controller actuating signal, *u*.

**Fig 6:**
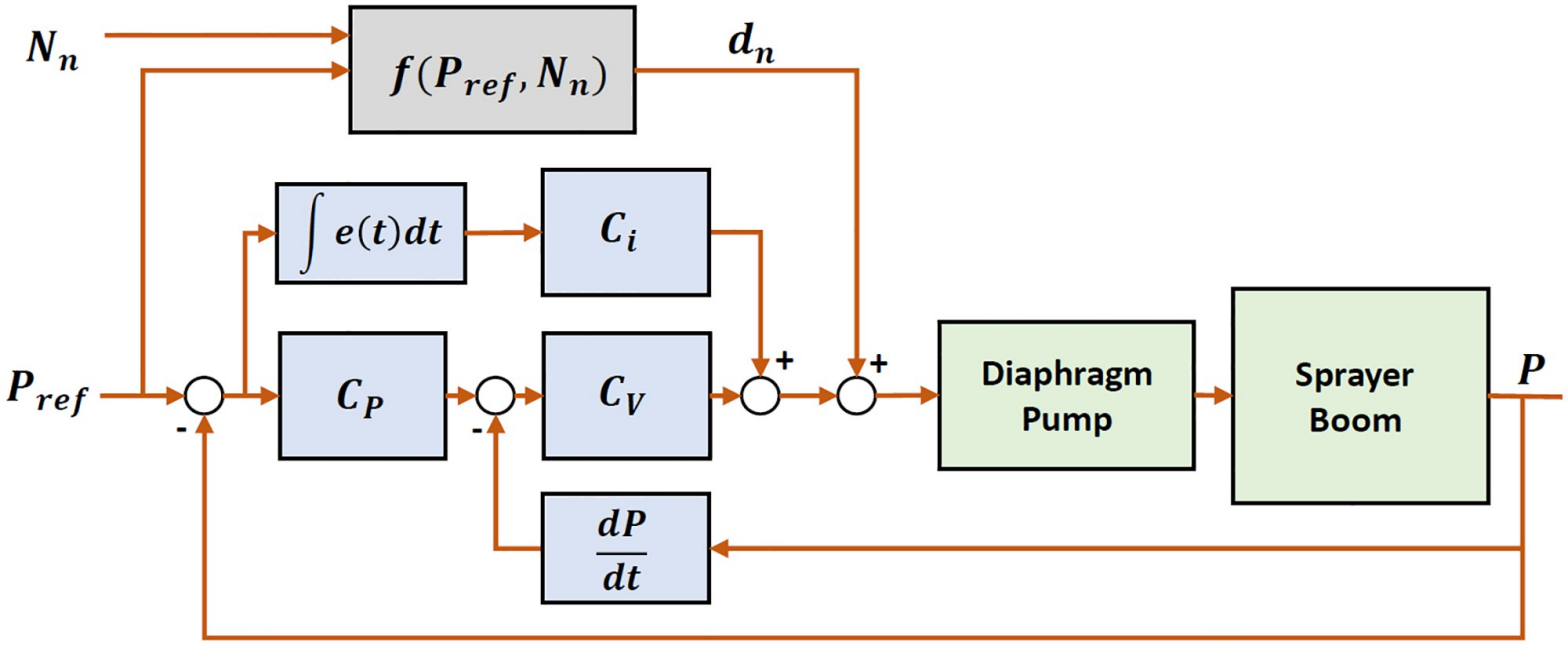
Block diagram of cascaded controller for pressure control system.

The magnitude of the five actuating signals measured in a steady state region of a close-loop pressure response when operated independently, i.e. without disturbance model. These measured values is then used to construct the disturbance function’s outputs. The number of active SVs, *N*_*n*_ operated in different combination are mentioned in Table 4. For each combination, the reference pressure was set to 20 psi and the corresponding steady state pressure, flow rate and the actuating signal as a PWM value were recorded. The disturbance function maps the strength of each actuation signal, *N*_*n*_, to the corresponding active number of solenoid valves, producing the output *d*_*n*_ that is fed forward and added to the control variable. This feedforward term generates an early control action before the main controller plays its role using the feedback information. As a result, the pressure disturbance can be lowered more quickly than if the cascaded controller’s control signal, *u*, was used alone. The complete control scheme of cascaded controller combined with the disturbance attenuation function can be seen in Fig 6.

**Table 4:** Pressure-flow response for open solenoid-valves.

Total Open SVs	SVs States: 1 = Open, 0 = Close					PWM (max = 255)	Flow Rate (l/m)	Pressure (psi)
	n_1_	n_2_	n_3_	n_4_	n_5_			
1	0	0	1	0	0	113	0.856	20.22
2	1	0	0	0	1	164	1.548	20.93
3	1	0	1	1	0	194	2.489	20.43
4	0	1	1	1	1	224	3.501	20.79
5	1	1	1	1	1	250	4.161	19.89

The response of disturbance attenuation function, *d*_*n*_ is added to the control action of the cascaded controller, *u*, the resultant control action, is expressed by the Eq (11).

$$\begin{array}{c} \begin{array}{c} u=f\left(P_{ref},N_{n}\right)+C_{v}\left(C_{p}\left(P_{ref}-P\right)-\dot{P}\right)+C_{i}\int \left(P_{ref}-P\right)dt \end{array} \end{array}$$ (11)

The effective demonstration of the proposed controller can be seen in result section, where the sprayer pressure control system, helped by the disturbance attenuation function, responds and settles more quickly (to the reference pressure).

There may occur an offset error in the steady-state response as these early action values are fixed feedforwarded values provided by the disturbance attenuation function. This offset value is then eliminated by the cascaded controller using the actual pressure, rate of change of pressure as feedback information, and the integrator action for eliminating the steady-state error.

## Experimental tests and procedure

**Fig 7:**
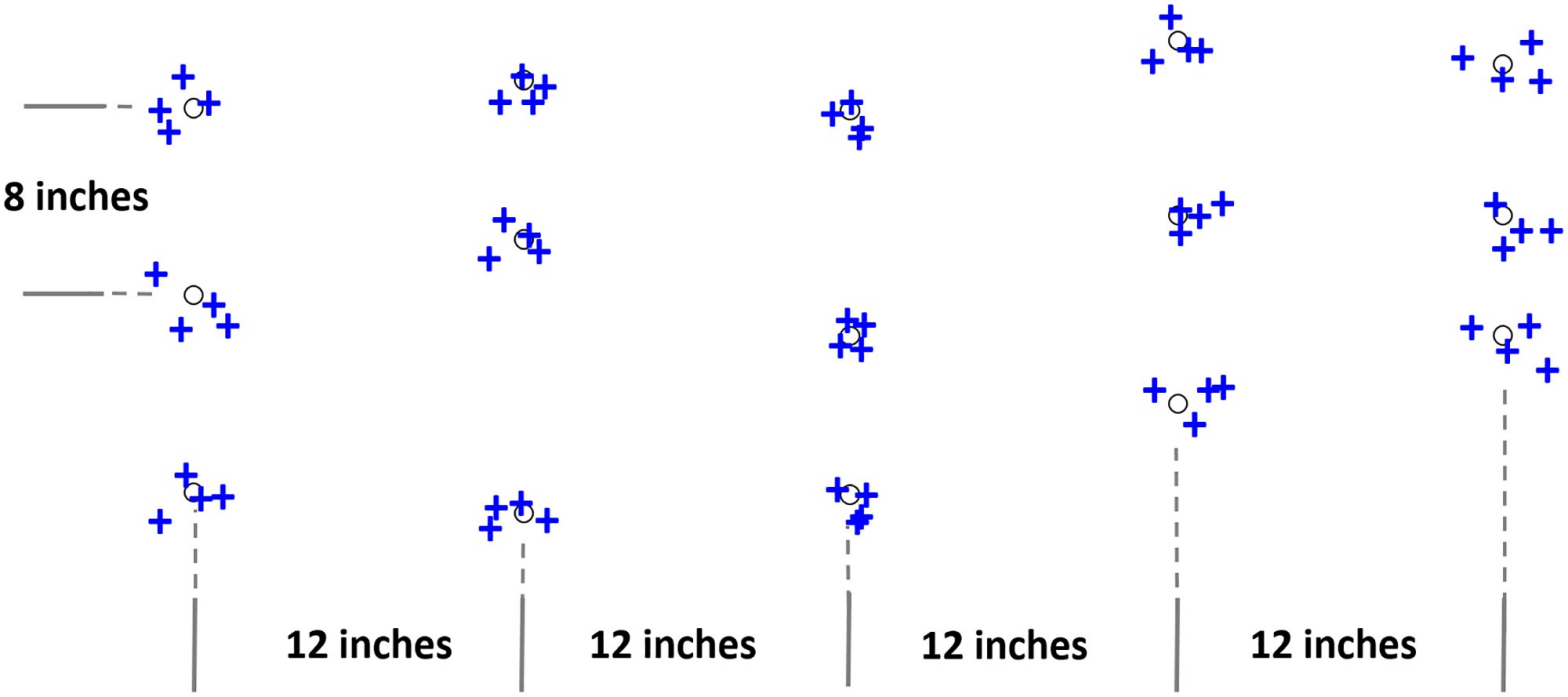
Approximate actual sprayed positions (plus sign) of the desired position (circle sign).

**Fig 8:**
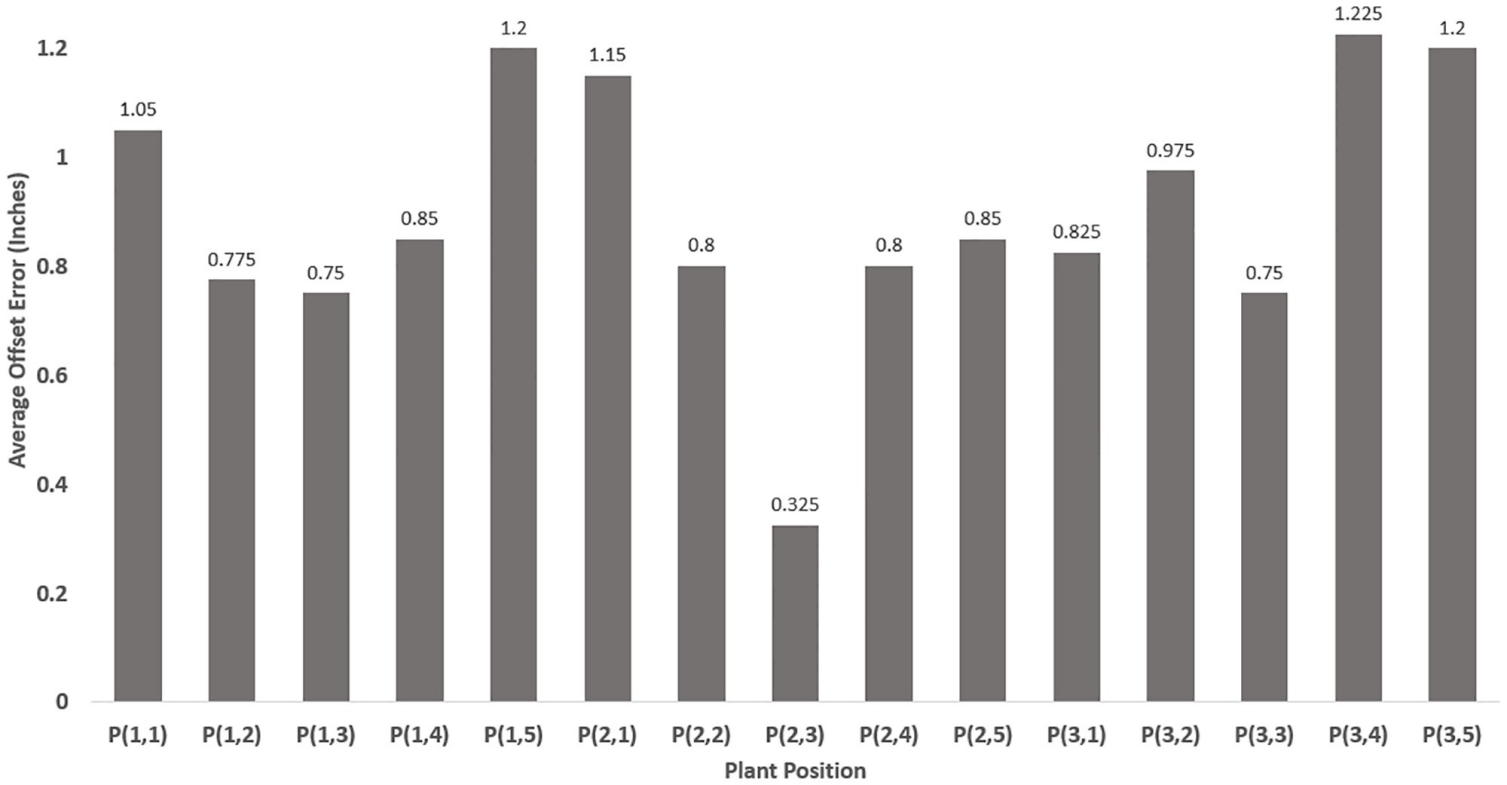
Average spray position error.

Before evaluating the spraying performance in outdoor real agricultural fields, preliminary tests were conducted in an indoor laboratory setting to test performance of the vision and spraying systems. First, different indoor trials were executed on synthetic plants under the same lighting conditions. While moving the robot in straight line following the middle row, the sprayed locations relative to the desired spraying location were recorded. Fig 7 depicts these as blue plus-signs and black circles, respectively. The average offset errors in positions of each plant are plotted as shown in Fig 8.

These inaccuracies are primarily caused by robot’s deviation from the middle row during navigation which was controlled remotely. Additionally, any unintentional adjustments to the camera’s orientation would result in proportional errors in the world frame along the *x*_*w*_ and *y*_*w*_ coordinates positions. The row following technique was used to address this issue and enable autonomous navigation on the robot. The application will eventually be made robust and adaptable to any farm layout or size using a Real Time Kinematics (RTK) GPS receiver aboard the robot with at least 1cm positional accuracy.

**Fig 9:**
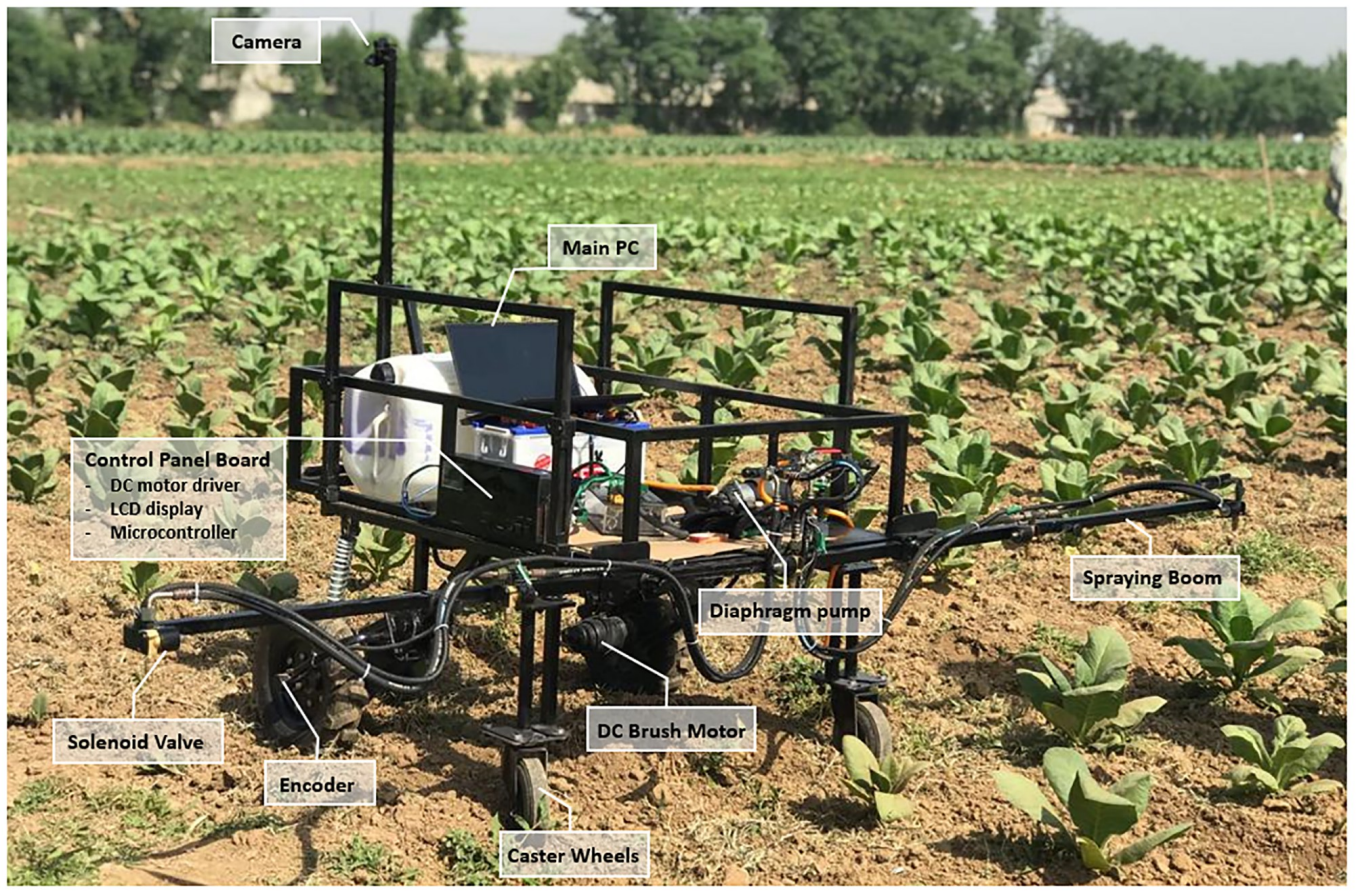
Field testing for selective spraying application.

In the final testing stage, the system performance is validated by conducting the real time experiments in the outdoor tobacco field Fig 9. As the weather conditions and the growth level of the tobacco seedling are the crucial parameters for the performance of the vision subsystem, we conducted our experiments at the most suitable time and field conditions.

### Results and discussion

The effectiveness of the object (tobacco) detection model can be assessed using a number of metrics. In this study, *F*_1_ score Eq (12), Precision Eq (13), and Recall Eq (14) were used. The *F*_1_ score, as proposed by Dice [42], represents the harmonic mean of precision and recall values. The Mean Average Precision (mAP) was also used as a performance metric as a measure of the average detection precision. It represents the area under the precision-recall curve at a defined value of IoU (e.g., mAP@50 represents the area under the precision-recall curve with a grade of overlapping bounding boxes of 50%). Both the test and validation datasets’ performance metrics were computed.

$$\begin{array}{c} \begin{array}{c} F_{1}=\frac{2R_{precision}R_{recall}}{R_{precision}+R_{recall}} \end{array} \end{array}$$ (12)

$$\begin{array}{c} \begin{array}{c} R_{precision}=\frac{T_{positive}}{T_{positive}+F_{positive}} \end{array} \end{array}$$ (13)

$$\begin{array}{c} \begin{array}{c} R_{recall}=\frac{T_{positive}}{T_{positive}+F_{negative}} \end{array} \end{array}$$ (14)

Here, the rates of precision and recall are denoted by *R*_*precision*_ and *R*_*recall*_, respectively, while the scores for true positives, false positives, and false negatives are denoted by *T*_*positive*_, *F*_*positive*_, and *F*_*negative*_, respectively.

#### Accuracy assessment on the validation dataset

A variety of trained deep learning models were used to classify the validation dataset, which consisted of 100 images of tobacco plant acquired under real field conditions. Comparisons were made using mAP@50 and the *F*1 score. In order to determine whether previously trained models could be used for real-time detection in the given application, each model’s detection speed was also assessed in frames per second (FPS). The *F*_1_-score and mAP@50 comparison results for objects (tobacco) in the pictures dataset are summarized in Table 5. Experimental results showed that YOLOv5n performed better than other models, as seen by the mAP%50 (91%), *F*_1_ (87.2%), and FPS (67) scores. With an FPS of 36, YOLOv5s is comparable but substantially slower in terms of processing speed.

**Table 5:** YOLO algorithms performance for tobacco crop detection.

Model	mAP@0.50	Recall	F_1_-score	FPS
YOLOv3-tiny	40.72%	24%	30%	226
YOLOv3	65.5%	65%	65.25%	30
YOLOv4-tiny	32.09%	40%	42%	222
YOLOv4	76.43%	60%	72%	29
YOLOv5n	91%	87.3%	87.2%	67
YOLOv5s	91.8%	88.3%	88.64%	36

**Fig 10:**
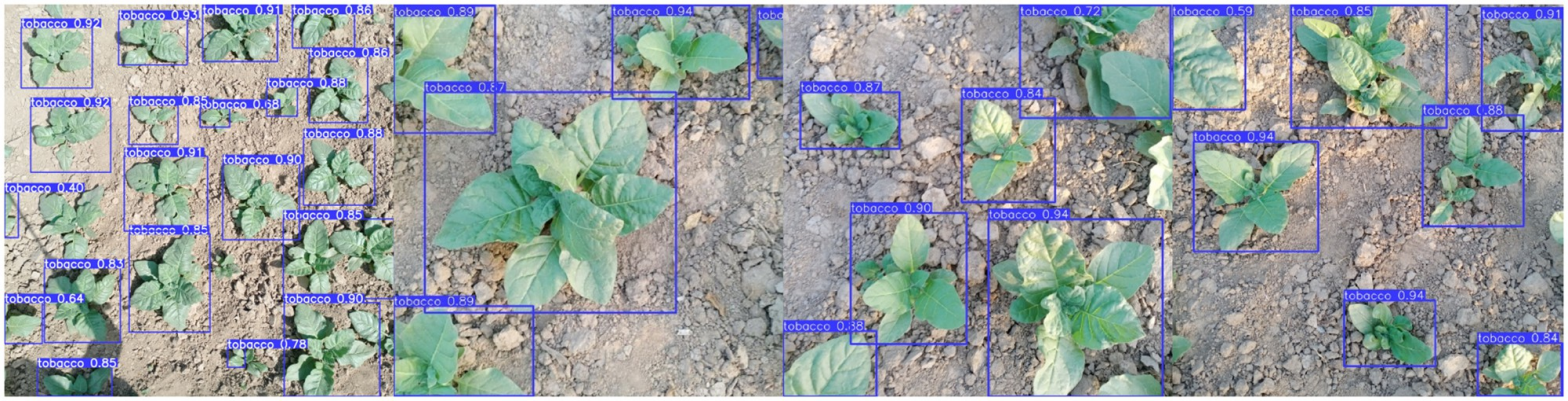
Detection of tobacco plant using YOLOv5n model.

The results that compare the inference time are also shown in Table 5. As shown, the average detection speed in FPS is 226, 30, 222, 29, 67 and 36 for YOLOv3-tiny, YOLOv3, YOLOv4-tiny, YOLOv4,YOLOv5n and YOLOv5s, respectively. For robots performing targeted spraying in agricultural fields at speeds between 2 and 10 kph, FPS values of 67 or above are sufficient. The results of tobacco crop detection in actual field conditions are shown in Fig 10.

**Fig 11:**
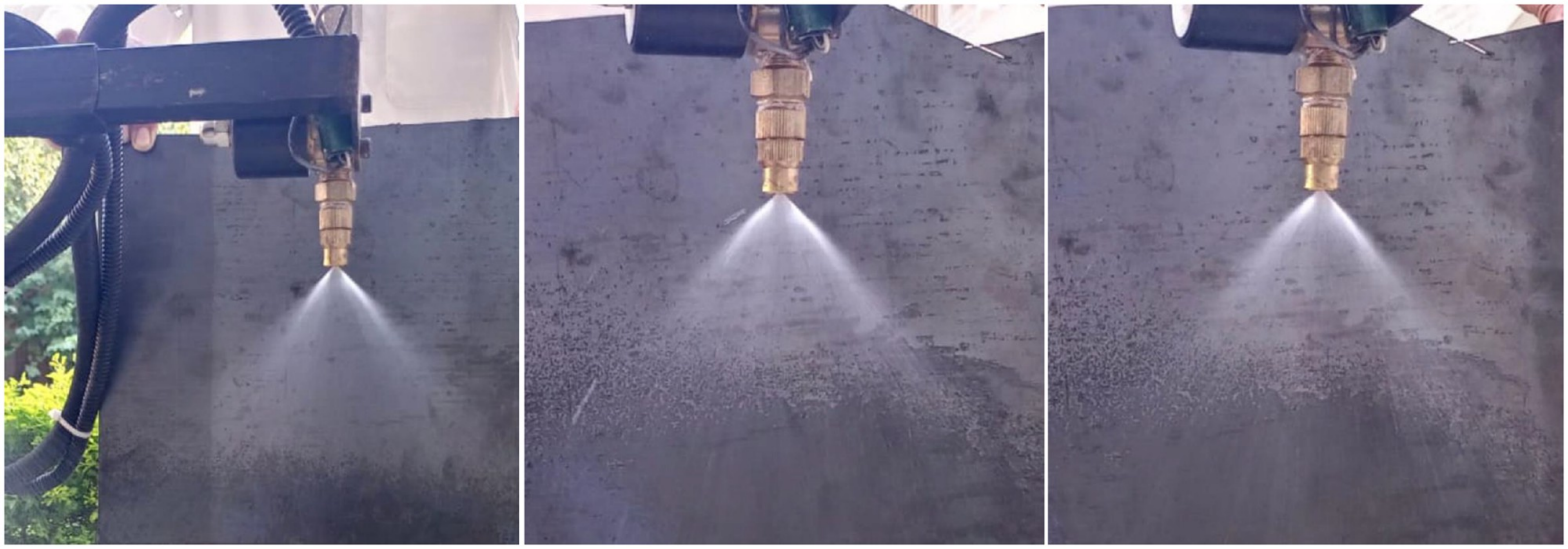
Constant spray angle achieved under different number of active solenoid valves at constant pressure.

The constant angle tests were conducted for verifying the qualitative nature of spraying under the constant pressure at 20 psi. Each image shown in Fig 11 presents a hollow cone nozzles making a hollow cone spray pattern with ring-shaped impact area. The angle of one solenoid-valve was measured under three different cases. In each case, under the close-loop pressure control system, the number of active solenoid-valves varied as *N*_*n*_ = 5, 4, 3 giving the same spraying angle of 80 degrees. This demonstrates how a spraying boom under constant pressure would deliver a consistent flow rate and droplet size at each nozzle.

**Fig 12:**
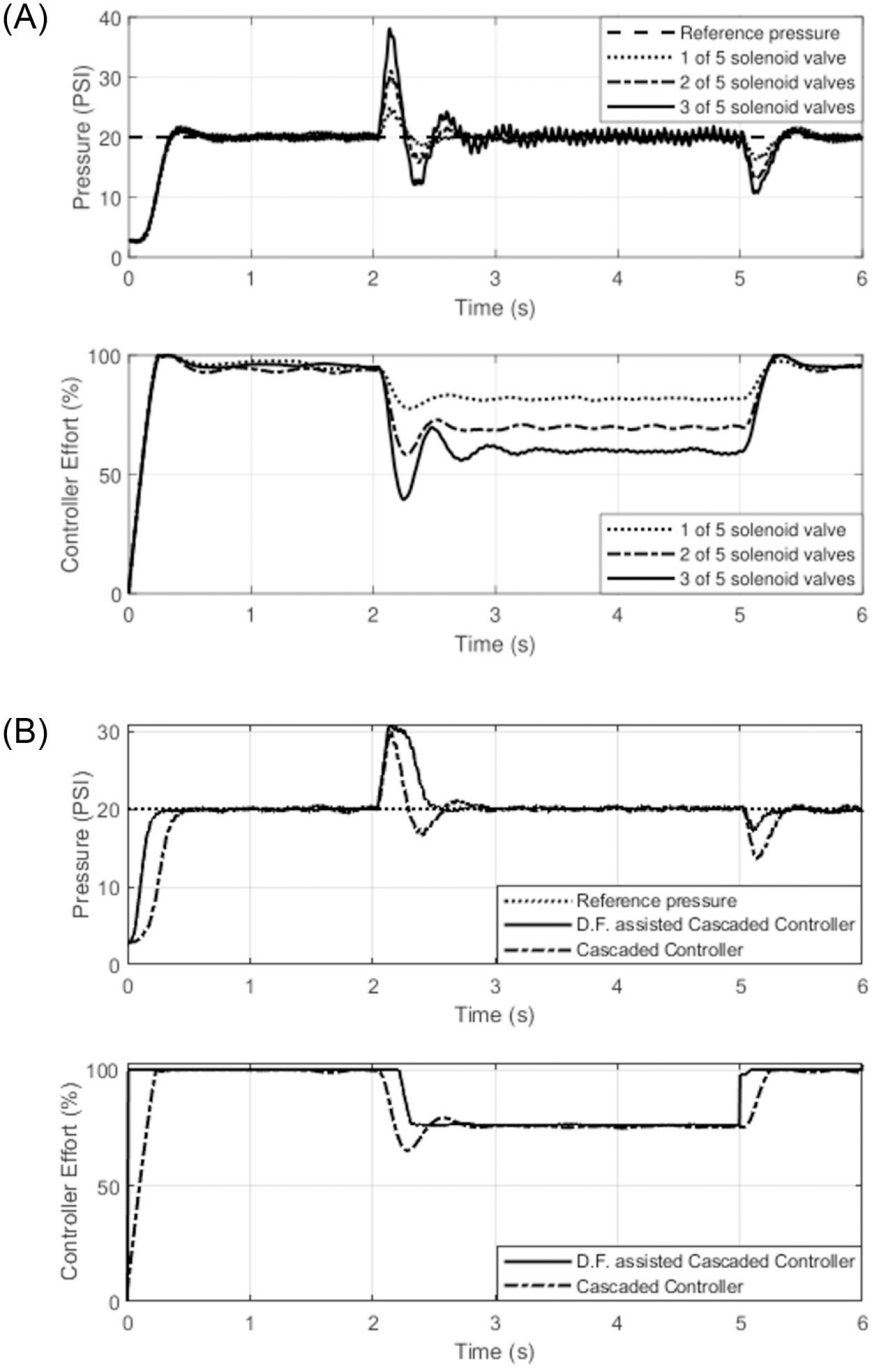
(**a**) Spray boom pressure response based on cascaded controller. (**b**) Cascaded vs cascaded assisted by disturbance attenuation function.

As shown in Fig 12a, the pressure reaches to steady state of 20 psi when all the 5 solenoid-valves were held open. The steady state pressure response was disturbed by instantaneously closing different numbers of solenoid-valves. In three different experiments, where 3 valves, 2 valves and 1 valve were closed at time 2 seconds, causing the corresponding pressure rises in the boom. Similar to this, the pressure decreased in the same manner when the same quantity of SVs were closed at time 5 seconds.

Similar response curves for cascaded and cascaded controllers assisted by the disturbance attenuation function are plotted in Fig 12b. It is evident that the cascaded controller reaches the steady state later than the cascaded controller when supported with disturbance attenuation function and exhibits a significant rise in transient state response. Additionally, it dampened the oscillations that were caused by the closure of two of the five solenoid valves at the 2 second mark in reaching the reference pressure. When the solenoid-valves open again at time 5, the amount of the perturbation is less than it would be with a cascaded controller working alone.

**Fig 13:**
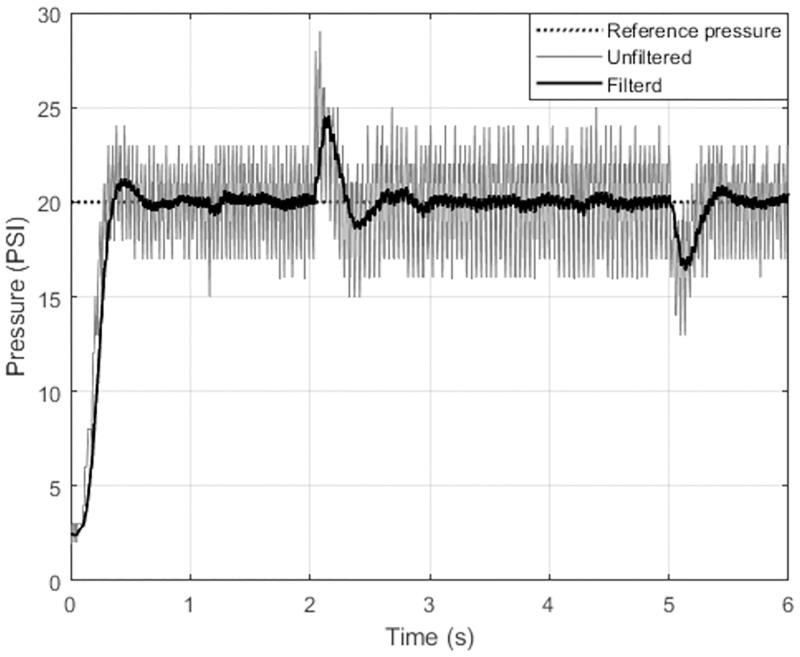
Filtered pressure signal using low-pass filter.

A low-pass filter is used to reduce noise in pressure measurements. The generated signal from the pressure measurement is converged using a low-pass filter, as illustrated in Fig 13 and the calculated Signal to Noise Ratio (SNR) is 11.4 db.

The efficiency of the pressure control system is estimated by exposing the system to disturbances and observing its reaction under these abnormal conditions using system’s performance indices or objective functions. The the tuning of the controller is performed using Ziegler and Nichols method and with the desired control performance parameters of no overshoot and less settling and rise time.

Table 6 summarizes the comparison in term of energy consumption between the cascaded controller and disturbance attenuation based cascaded controller (Cascaded Controller assisted with Disturbance Attenuation Function i-e D.F. assisted Cascaded controller). These performances are expressed in terms of objective functions such as Integrated Squared Error (ISE), Integrated Absolute Error (IAE), Integrated Time Squared Error, and Integrated Time Absolute Error (IATE).

**Table 6:** Objective function for pressure control system.

Controller	ISE	IAE	ITSE	IATE
Cascaded Controller	13386.85	1386.03	9645.45	2199.08
D.F. assisted Cascaded Controller	8927.57	1014.52	9704.20	1765.91

Where as, Table 7 shows the pressure control system stability performance parameters for both the cascaded controller and disturbance attenuation based cascaded controller. The stability analysis are performed with the ISE objective function and their corresponding energies are determined. The performance efficiency of the cascaded controller assisted with disturbance attenuation function is higher than the standalone cascaded controller in terms of disturbance rejection and less energy consumption.

**Table 7:** Stability analysis of pressure system with ISE objective function.

Controller	Rise time (s)	Settling time (s)	% Overshoot	ISE
Cascaded Controller	0.26	0.37	0.2	13386.85
D.F. assisted Cascaded Controller	0.1	0.2	0	8927.57

## Conclusion

This paper describes the development of a deep learning-based robotic solution for targeted spraying in precision agriculture. Two key contributions have been made based on real field experimentation: (1) an experimental comparison and performance assessment of YOLO-based deep learning models for detecting tobacco crop; and (2) a cascaded pressure control method to achieve the ideal dose of pesticide application during selective spraying. Using distance measurements from the wheel position encoders, the robotic solution additionally synchronizes image acquisition, object detection, and spraying in real time. The experiments concluded that the results generated by YOLO5n model were more convincing than its other four versions in terms of its 87% *F*_1_-score and 67 FPS rate. Moreover, a closed-loop control system is designed to keep the pressure constant in the sprayer’s fluid circuit. The pressure control system performance was further improved by assisting the close-loop system with a disturbance attenuation function. The targeted application of fertilizers [43], herbicides (to kill weeds), and insecticides are the planned future extensions of this work.
